# Supplementary figures and images for: Satellite DNA-containing gigantic introns in a unique gene expression program during Drosophila spermatogenesis
Source: PLoS Genet. 2019 May 9;15(5):e1008028. doi: 10.1371/journal.pgen.1008028 (PMC6508621; doi:10.1371/journal.pgen.1008028)

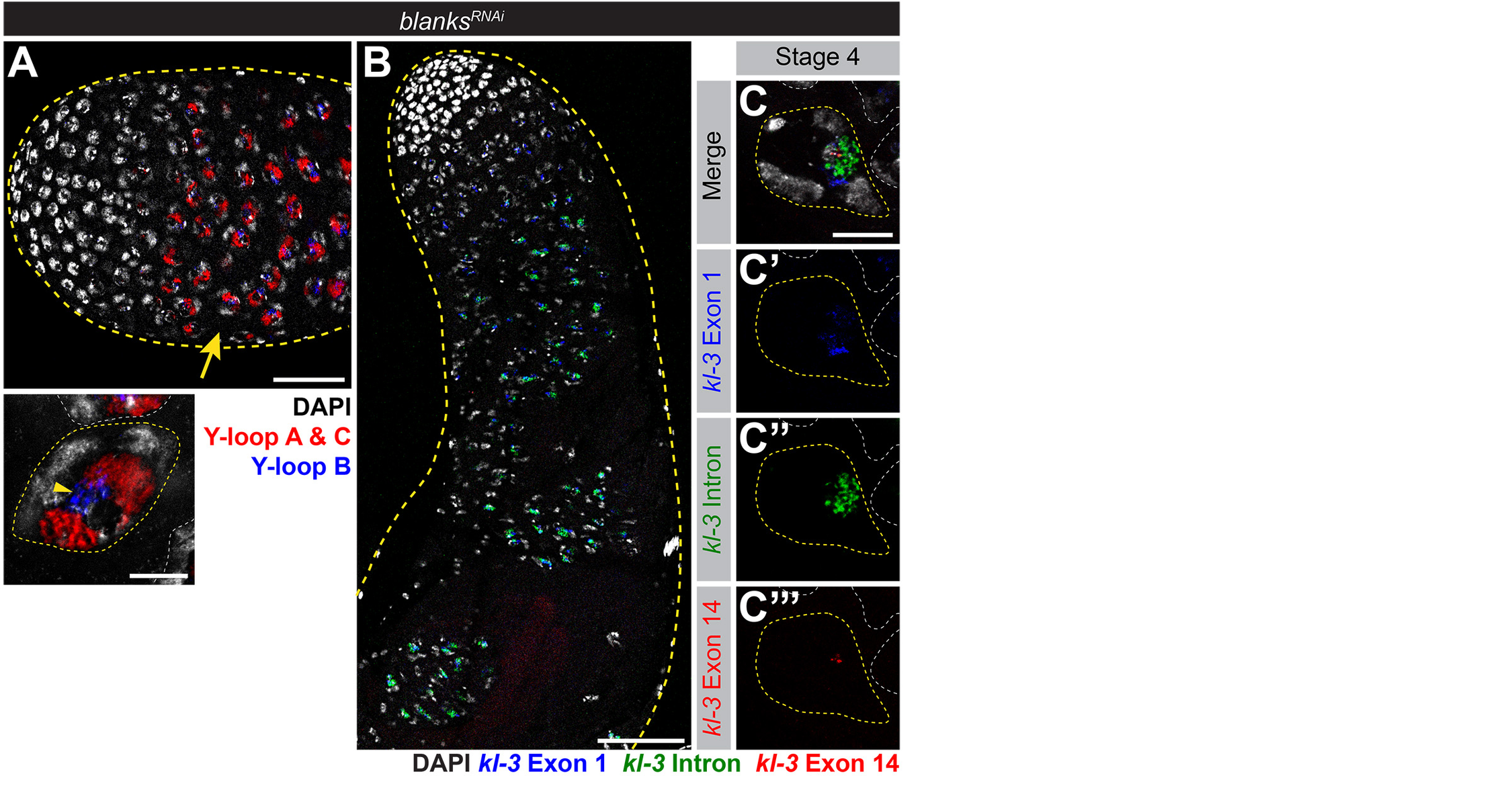

Supplement: S1 Fig — (A) RNA FISH against the Y-loop gene intronic transcripts in bam-gal4>UAS-blanksTRiP.HMS00078 testes. Testis outline (yellow dashed line), Y-loops A and C (Cy3-(AAGAC)6, red), Y-loop B (Cy5-(AATAT)6, blue) and DAPI (white). Comparable stage SC (yellow arrow, compare to Fig 3A and 3B). Bar: 50μm. High magnification image of a single SC at a comparable stage (compare to Fig 3A and 3B) is provided below. SC nucleus (yellow dashed line) and nuclei of neighboring cells (white dashed line). Bar: 10μm. (B, C) RNA FISH against kl-3 in bam-gal4>UAS-blanksTRiP.HMS00078 testes. Exon 1 (blue), kl-3 intron (Alexa488-(AATAT)6, green), Exon 14 (red) and DAPI (white). (B) Apical third of the testis through the end of SC development (yellow dashed line). Bar: 75μm. (C) Single late SC nucleus (yellow dashed line). Nuclei of neighboring cells (white dashed line) and mRNA granules (yellow arrows). Bar: 10μm. (TIF) [file pgen.1008028.s001.tif]

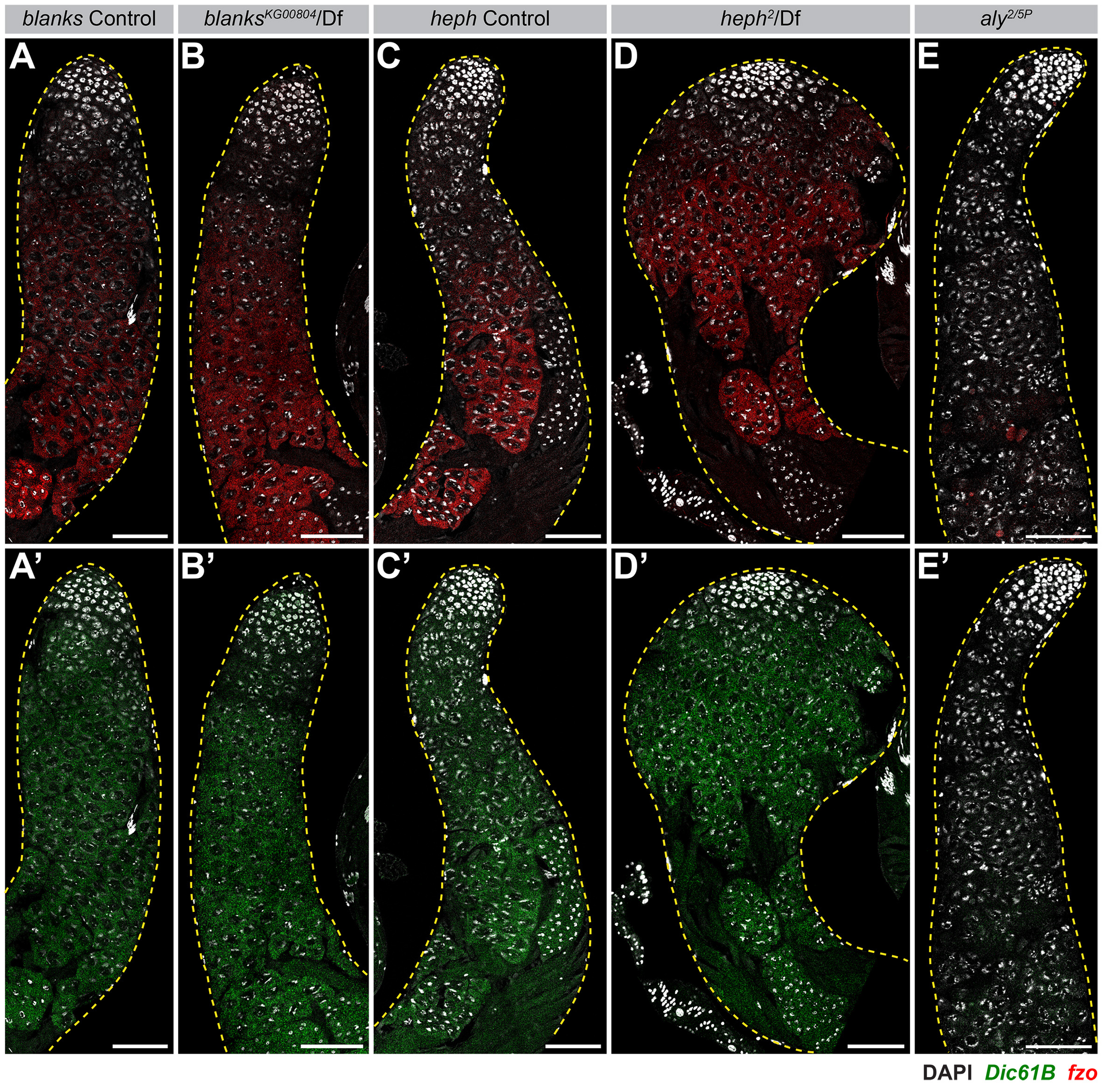

Supplement: S2 Fig — RNA FISH against fzo (A-E) and Dic61B (A’-E’) in blanks controls (A), blanksKG00084/Df (B), heph controls (C), heph2/Df (D), and aly2/5P (E). Apical third of the testis through the end of SC development (yellow dashed line). DAPI (white). Bar: 75μm. (TIF) [file pgen.1008028.s002.tif]

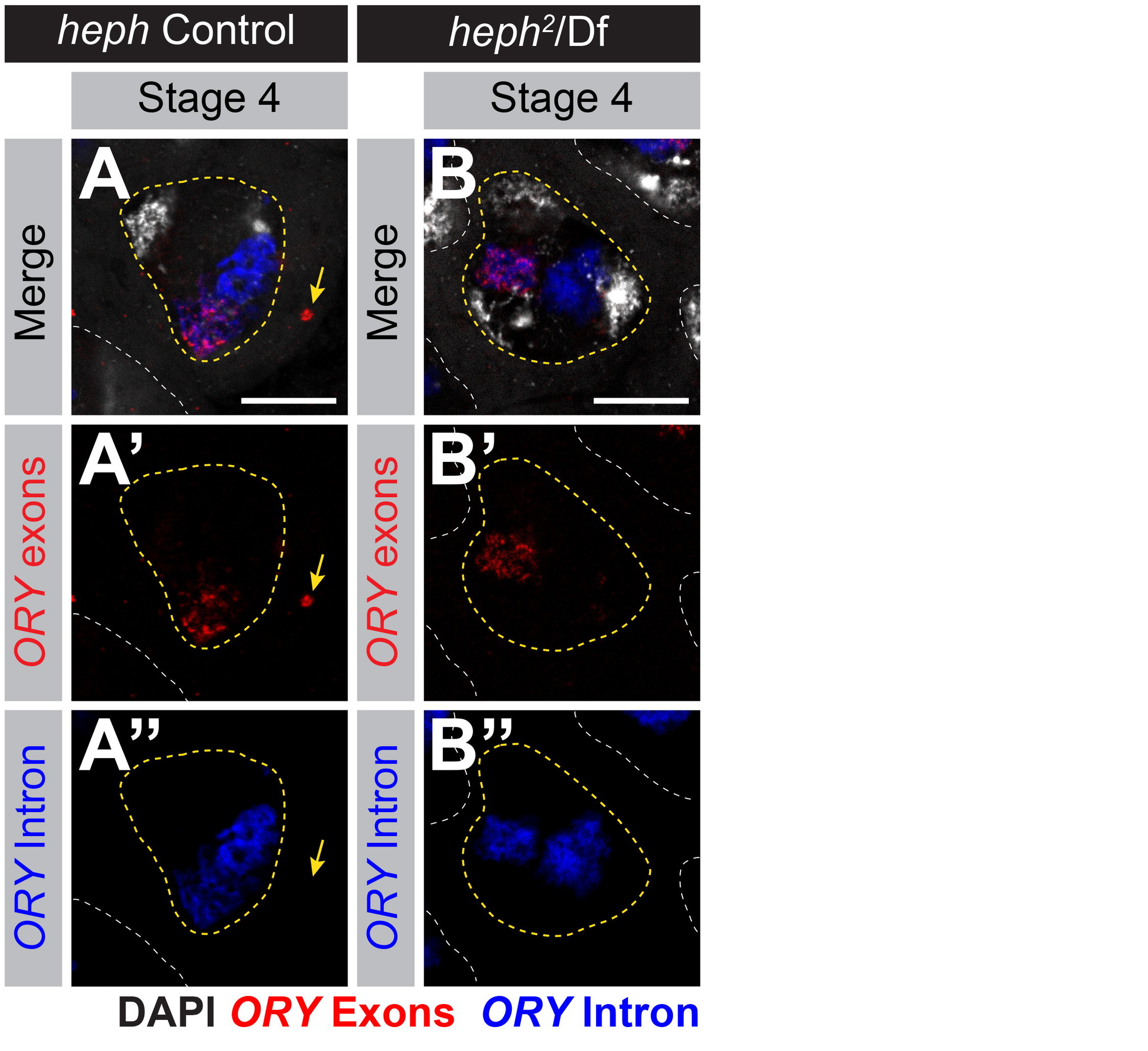

Supplement: S3 Fig — RNA FISH against ORY in heph controls (A) and heph2/Df (B). Exons (red), ORY intron (Cy3-(AAGAC)6, blue), DAPI (white), single late SC nucleus (yellow dashed line), nuclei of neighboring cells (white dashed line) and mRNA granules (yellow arrows). Bar: 10μm. (TIF) [file pgen.1008028.s003.tif]
