# Supplementary material for: Satellite DNA-containing gigantic introns in a unique gene expression program during Drosophila spermatogenesis
Source: PLoS Genet. 2019 May 9;15(5):e1008028. doi: 10.1371/journal.pgen.1008028 (PMC6508621; doi:10.1371/journal.pgen.1008028)
Supplement: S1 File — (DOCX) [file pgen.1008028.s004.docx]

**S1 File: Probes for RNA FISH**

**Probes targeted against satellite repeat transcripts**

| **Probe Target** | **5’-Sequence-3’** |
| --- | --- |
| (AATAT)n | Alexa488-ATATTATATTATATTATATTATATTATATT  Cy5-ATATTATATTATATTATATTATATTATATT |
| (AAGAC)n | Cy3-AAGACAAGACAAGACAAGACAAGACAAGAC  Cy5-AAGACAAGACAAGACAAGACAAGACAAGAC |

**Stellaris RNA FISH probe sets**

| **Probe Target** | **Dye** | **5’-Sequence-3’-Dye** |
| --- | --- | --- |
| *kl-3*, Exon 1 | Quasar® 670 | taacattcctttctggatcc, cgcgaaacgccaaagagttt, gcagcacgctttaacatgtt, ttggtcacttacactaggtc, tatcgtcttctttgttggtc, cctcatttctcgaagtaact, caggcttgaaatccgttgtt, aaacataccgctggtttggg, atacccaacaaatctgcaca, gttactatttcctcaggatc, ttgctttcatccacaatacc, accatttacattctcaacat, gggacctttttcctcaaata, cgttacttatcattatggcc, cggaatcagttggatatcct, tttaagcttttcctggtagc, agagcgttgaatttcagtgt, actagatccgacctcaaaca, gtcgatatacaacagtccac, accgaacggttatcaatcga, aaatattgccacctcatcac, aagcaagagtttcgctcttc, cggctttaaaacgtgatcca, caaattcggtcacagcttct, tgagtttgctctttttctgc, acagttgcttcagatgattt, cctttaaatagttcatgcga, tttgccatctcacaagtaat, gtatttttgaactggcttca, caaccagtcgaactcgtgta, gccattgctcaaaataacgt, gtataccttgaatttgtcga, gtatttgttttccctctact, ctacatcgggtgtatctctt, ccaattgaccaacatttgca, tatatctggccaacatacgc, gtaacaaactccgttatggt, gtggttattaaatgctcggg, agataatgtcaagcaatcct |
| *kl-3*, Exon 14 | Quasar® 570 | gtactttgacatagccatgg, aagatttgcctttaagggca, tgatatttagcctcttgcac, ctgcttcttgtagatcactt, cgttttctttttgttgcagt, tttttggcctcgtctaatac, aaccaccaataagagcggtt, ttcagtccatcggatttttt, cggtcggtctcacttttaaa, ggagaataacatctccgacc, tcttgattaaatggtcccgt, atgttccactcaccaatttg, ttgacagttcatctgttggt, ttttaatccacacttttccc, taatcggaattcccatgcta, taactcctctgcaacatctt, aaggttgtccaagcaaggat, tccaatccacttctttatca, gattgggtagctttgttgta, cgcgcaaatatttcaggtgt, ccacgcattgtcacagtaaa, gaacccgttcatcttctaat, tttcatgtttccagtcacag, ttcctttcgtagtggacaac, cctcaatcactgtaacgtcg, tccttaacttcaatggcagt, cagcgtttatttttgcttct, acactacctcttgtagcaac, gcatcaaagcgctcaaggaa, tcctctagatttgtagcgat, ttaactcaagctggcgatca, cgcaccgccttttataaata, caccgaaacggaacaggagg, tataccacgtaaaccaagcc, ccatacaccacgaacgaaca, agtttagtactactggctcg, aatcattggcataagttccc, agctcattttttttggcgag, cttggcccatagaaatagga, cgtcttctaggcaggataat, tcctaagtgacagttttgca, actgtaagctctaccatgta, agcaggtggttcattagtat, tttttagtccagcacgtatt, tggagattgcgaatagtcca, tgcataccagtcggatgaat, tcctgctccttaaaactgta, cgtttgccatgtcatcaata |
| *kl-5*, Exons 1-6 | Quasar® 570 | cttcttttccttttcgtcag, aaaaactccggacggttgtc, ttgtcttggttaggtagttc, ccacttatccagcttaagac, cctaaactcgttggttgtta, atacgtttcttgttgggatt, ccaccggaattgattgtgaa, ctggaaagctgtaggatgga, agcaactttataccgtggtc, gaagtggtgttaagtaccga, atttgctaatgggttcggta, atcccacttgatttactgtg, tctgtcttcatatcgtttgc, tccatttcgcatttcttgag, aacgagacctttcatctggg, catgacttcgtccatgcaaa, gcttcataagagggttaacc, caagccactttacaaccata, tttacgaggtcttcaacgga, tgcctctggtagtggaaaat, caagttctcaaggttttcca, agtctttatacgcctatctc, tctgcgataagaatgctcga, acttagttatgtctctagcc, gtccatatcgtttgtttcaa, gtcgtatatctgatcggttc, cactaatccaattgtcagca, tgaaaataccgcgagtgacc, gttctgttgacacagtcgat, ggaatatggagtctggttct, ttataggcttcgtcgacatc, tgtaatactctaggtgctgc, cagtcctgcgagttaaatgt, atcgtccgaatatttcatcc, tcttttagctcttccaatcg, ccccataacaattttttcca, aagggcgaaagttatctgcc, tgctgttgtactcttcaaga, aatatttgtccactcacggt, acagagaattttctgcgtcc, tggccttgaagcttaaacga, tgaaacgataccatgcgctc, atgtgtgtcttcaagacctt, gcgaagaagtaaggagcctg, gggttcgacatgttttttga, atcatccacaatgacatgca, taacgcacatgatctcttcc, cgagcgccgtaaaaacgttt |
| *kl-5*, Exons 16-17 | Fluorescein-C3 | ttgtggtccgaatttcctac, taaacgggtagctacggttc, tgatattgtcaaatcgccca, ccaggtaattgtacagcaca, caaggtactctggtattggc, ccatacataatctctccgaa, ccagtcgtctgtaatgtgac, gataagttcggcataagcgg, agctctggctgcataaattc, aaaccctggcaatactctag, atttaagtattcctggagcc, atgtaattgtggtagccagt, gagatggactttcagacgga, gcatttgaatgaaggccgta, cgtagttaggaacccaatct, attcggaagagtcgttcaga, tctcggctgcaactcgaaaa, aaactgtctcaccaccacta, gttttttataatgtcttcct, aatggtgtgggagttttgtc, cgcgacccattagttctaaa, atgtatggacttcggtcttc, cgctcacactcttgaaatgc, gcttcaattcagtcataaga, aggtcaagctcattcagaga, agtcaattctcctttaaggc, ccattaaatcctccataact, gcacttggtccatgtaaaga, aaccaggattgtagccctag, cagccgcaacattaaatcgg, aggcatgcgaaagtcggcaa, atcctgccaaccaaattgat, aggagcgactgaggattaaa, cgtctgttgcatgatagctg, gacacattctatcgagaggc, ttccactttttggtgacatc, ttgatataggcaccctctcg, tgctccctccatgaaaagac, aatggttcccattttcatgt, gttcctttaaaaaggcgtct, agaacaggcatagcaggaaa, cttgggttactgcctttatg, gacatttttaatatcctgct, cggattttgtaaactgggca, caaacgaaggtgggtcctcg, ctctcggcttttcaagttaa, ctagagtccacttacttgct, tgcaagagaagacaaacccc |
| *ks-1 (ORY)*, All exons | Quasar® 670 | tttttggctttctttctgtc, aaaagttgaggctccgagtt, cgtttaattcgcgatgcttc, cttcatctacataccgacga, tccgatgttgaagtcagttc, cccccaacaaatctttaagt, tgatgcatctgattctttcc, tcttgcactaactgttctcg, cagttgtaccactatttcgg, ttagctcgagaagccatttt, attcaacgtttaggcgttcg, ccattgtcttcatcaaagct, tttccatgtgcttctttttg, ctctattcgcaatatccagt, agtttacgtgtcgtttctga, gcattgccttatttaatgcg, gccttaactgctttatcatt, aagcctgctcgttaattgag, ttgtacattcttgttgtcgc, catccttctccagagaatta, gacgaatatcatccgttcgg, cacgttgcaatgtctctttg, atgttttaagtccgccatag, tctttctctgctttggattt, cgtcttcagagagatttggt, tcgtgtaacttttccgtgag, acggactccaactgtttttt, cgctgaagcatcattttctc, ttttacttcgtttgcgctta, gccactaagttttctttgtt, tttctctcatatccttacgt, caatccgactaggttacgtt, aacaatctcgtcattccgtc, gggcattttgtgcaatttga, aaggcattcactttcagtgt, ctcatgtcagcagtactttt, tcttcagttagagctcgtat, caacgatgtactcctgtagg, aatttcttaggatcttcgcc, tattgactgctttagggagc, agcctcacacagtttatttt, gcatatgagatagcatcctt, taatgaacgctctgctgcta, gtcttgatttaagttccacc, aattgacagctctccgattt, tatttcgtttttcccatctg, atccacattccatatactct, atatccgttaacttcgcaca |
| *fzo*, All Exons | Quasar® 570 | aaacgaggacaccgacgacg, gcgaagacgacgatgaggat, gcgtccacaaactcacttaa, tatatatcctgcagttctgt, tttaacaggacagtctcctc, aaagaacctttgcaatggcc, gtccaaaaaatgccaccttc, cattgatcacggcacttttt, gcaggattttttcatgcaga, aaaacagctggtggtatggc, cagagttgagggttttaggg, atccaatatcgagcaacggt, tagctatctaggcaatcgtc, actcggcgttgagaactaga, ttgaagaactgcctttccac, agagatttggacgcgagagt, cccatcgattgttgagtata, caacgttccatatgctgatc, acacctaattcatccacgag, aacatgatagatcctttccc, cgtaatctgaccattcctta, gaaactcctgatatcgctgt, accgcgaggcaattcgaaaa, acttagcaagtgtggaccaa, ctctatttcggcattcaagt, tctatcctcttgcattagta, gtcatttcagtcagttcttc, tctggtcgttcaaaacgcac, gatagcacagacgagggtat, ctgggaattccgggtgaaat, ataacgagcgttggtactgg, ggggtatggagagacattga, aagcccaatttctttctcta, tagattagttgccaatcgca, tctggctgaaagtcactcat, ttcgaaatggacttgcgtgc, tccgttaactaaaggtggca, ttcaccaaagattccagcat, tcagtaaaacggtgcccaag, ccagttgaacgaacggatgg, cccaccaaggatcaatacaa, cttgaaacttcgctcttggg, ctaacagtttgctgcacatc, gtcgcaactggagctcaaaa, attcagtgtctcattggact, tgtatttgtttggtcagctc, gaaacttcttcaggctgagc, taggtctcttgaaagtctcc |
| *Dic61B*, Exon 4 (isoform A) | Quasar® 670 | gttcccagttaatagtttca, cttcgagagcctcaacagtg, aaggacttggtatacggctt, gacttgcgtaaggtgatctt, gtctggctgtgaatttcgaa, cagatactcgtagtttcggt, aattttgcctttccctattg, tctgagcgtcattgtctgag, gagtggtgaagagcagagta, ggttatgaggatggtgttga, aacgtaagagctgacggtgg, gcgtgtcgtacatctcaaag, actccttggtgatattgttc, cgaactcatagtggctgttg, tcgtcgtcctcaaaggtatc, cttgtctttttcggcaacgg, cggaacaggcgctctatctg, atgatggcgttgcgaaactc, tcgtttgtgttgctcgagag, ggtcaaagttacggaagcgt, catcgagtagacgtactcgg, taccagccgaaagagcaagt, cggcagaaatcaatgtcgct, tgagtacagtccgtaggcta, cggtacgtgctgtgaggaaa, acaggaaaacgctgccggag, ctcgcctggatttttaatgc, acatcgtagtagtatgcgcg, aagtttattgccgttactgg, aaggatggcaagaatggcga, tcgtacagaccaatggcgag, tgcaaactgctcacatctcg, aaacttgtttgatccagcgg, tgtctgtttcatgatcgtcg, gagaggctcaggaatggatc, tgataatccggaagcgggtg, aaccaagcaggaagggactt, actcgctccagaatcatttg, gaacgaaaatgccctcagga, ttagactcgcaggggatacg, gtagtgatgcttaagccttg, atgtccttgtgcagaggatg, tcatctgtcagcacgtagta, gattgagcacttgtggatgc, ccaagtactgatgctgataa, cgtcgtgacaacgcaggact, gaactccattacggtgacac, aggtgaggaagagtttggga |
