# Supplementary material for: Satellite DNA-containing gigantic introns in a unique gene expression program during Drosophila spermatogenesis
Source: PLoS Genet. 2019 May 9;15(5):e1008028. doi: 10.1371/journal.pgen.1008028 (PMC6508621; doi:10.1371/journal.pgen.1008028)
Supplement: S2 File — (DOCX) [file pgen.1008028.s005.docx]

**dsafdsaS2 File: Screen Summary**

| **Candidate Protein** | **Known Function/ Reason Selected^1^** | **Localization in Testis^2^** | **RNAi/Mutant Phenotype(s)^3^** |
| --- | --- | --- | --- |
| ADD domain-containing protein 1  (ADD1) | **GO:** heterochromatin^4^ | Germ cell DNA  (*Mi(PT-GFSTF.1)ADD1^MI09552-GFSTF.1^*) | ND |
| Always early  (Aly) | **GO:** regulation of gene expression, spermatid development, spermatogenesis  High testis expression  Male sterile, SC transcriptional program member^5^ [1, 2] | Previous studies: SC DNA, nucleolar periphery [3, 4] | SC arrest, little-to-no Y-loop gene transcript detected  (*aly^2/5P^*, gifts from Minx Fuller) |
| Argonaute-1  (AGO1) | **GO:** negative regulation of translation^6^, ribonucleoprotein complex | No signal  (*P(PTT-GA)AGO1^CA06914^*) | ND |
| Arrest  (Aret) | **GO:** germ cell development, mRNA binding, negative regulation of translation, P granule^7^  Male sterile [5]  RNA Recognition motif (RRM) domains | Spermatid cysts  (*Mi(PT-GFSTF.1)aret^MI00135-GFSTF.1^*) | Y-loop gene transcription normal, sterility^8^  (*P(PZ)aret^01284^*) |
| Beethoven  (Btv) | **GO:** axoneme, cilium assembly, cytoplasmic dynein complex, motile cilium | No signal  (*Mi(PT-GFSTF.0)btv^MI08510-GFSTF.0^*) | No phenotype detected  (*UAS*-*btv^TRiP.JF03010^*) |
| Belle  (Bel) | **GO:** regulation of gene expression, P granule spermatogenesis  High testis expression  Male sterile [6] | Cytoplasmic  (*P(PTT-GC)bel^CC00869^*) | Y-loop gene transcription normal, sterility, sperm head scattering  (*UAS*-*bel^TRiP.JF02884^*, *UAS*-*bel^TRiP.GL00057^*)  No phenotype detected  (*UAS-bel^TRiP.GL00205^*) |
| Bicaudal D  (BicD) | **GO:** intracellular mRNA localization^9^, mRNA transport | ND | Minor scattering of sperm heads  (*BicD^r5^*, *UAS-BicD^TRiP.HM05057^, UAS-BicD^TRiP.GL00325^, UAS-BicD^TRiP.HMS02622^*) |
| Blanks | **GO:** heterochromatin,  sperm individualization  High testis expression  Male sterile [7, 8]  Double stranded RNA binding domains | Y-loop  (*GFP-blanks*, Gift from Dean Smith) | *kl-3* transcriptional defects, sperm head scattering, sterility  (*P(SUPor-P)blanks^KG00804^*, *UAS-blanks^TRiP.HMS00078^*) |
| Boule  (Bol) | **GO:** mRNA binding, regulation of translation, spermatogenesis  High testis expression  Male sterile [9]  RRM domain | SC cytoplasm, spermatid cysts  (*Mi(PT-GFSTF.1)bol^MI00386-GFSTF.1^*)  Previous studies: Y-loop [10, 11] | Spermatid arrest, Y-loop gene transcription normal  (*P(PZ)bol^1^*) |
| Bruno 3  (Bru3) | **GO:** mRNA binding, negative regulation of translation, ribonucleoprotein complex  RRM domains | No signal  (*Mi(PT-GFSTF.0)bru3^MI02379-GFSTF.0^*) | ND |
| Caper | **GO:** mRNA binding, mRNA splicing  RRM domains | DNA  (*P(PTT-GC)Caper^CC01391^*) | ND |
| CG10845 | High testis expression  Kinesin^10^ | ND | Minor scattering of sperm heads  (*P(GD7107)v27320, P(KK107042)VIE-260B*) |
| CG12493 | **GO:** double-stranded RNA binding  High testis expression  Double-stranded RNA binding domain  Paralog of blanks [7] | ND | No phenotype  (*UAS-CG12493^TRiP.GL01161^*) |
| CG13901 | High testis expression | ND | Y-loop gene transcription largely normal, possible reduction in *kl-3* transcript, sterility  (*UAS-CG13901^TRiP.HMC06270^*) |
| CG3339 | **GO:** axoneme, cilium movement, dynein complex  Axonemal dynein heavy chain | No signal  (*Mi(PT-GFSTF.2)CG3339^MI00332-GFSTF.2^*) | ND |
| CG6254 | **GO:** negative regulation of transcription, nucleic acid binding  6 zinc finger domains | No signal  (*CG6254-GFP.FPTB*) | ND |
| CG7185 | **GO:** mRNA binding  RRM domain | DNA  (*P(PTT-GC)CG7185^CC00645^*) | ND |
| CG9492 | **GO:** cilium movement, dynein complex  Axonemal dynein heavy chain | No signal  (*Mi(PT-GFSTF.1)CG9492^MI09168-GFSTF.1^*) | ND |
| Clueless  (Clu) | **GO:** mRNA binding  Male sterile [12] | Cytoplasmic  (*P(PTT-GA)clu^G00271^*) | ND |
| Cut up  (Ctp) | **GO:** dynein complex, sperm individualization,  spermatogenesis  Male sterile [13] | Previous study: elongating spermatid cysts [13] | No phenotype  (*UAS-ctp^TRiP.HMS02760^*)  *kl-5* transcription defect, sperm head scattering, sterility  (*UAS-ctp^TRiP.HMS02554^*) |
| Double fault  (Dbf) | **GO:** chromosome organization, spermatogenesis  Male sterile, Y-loop defects [14] | ND | Possible reduced *kl-3* transcript levels in SCs, sperm head scattering, sterility (*P(PZ)dbf^1^*) |
| Dynein intermediate chain at 61B  (Dic61B) | **GO:** axonemal dynein complex, cilium movement, spermatogenesis  Testis expression  Male sterile [15, 16] | ND | Y-loop gene transcription normal, sperm head scattering, sterility  (*UAS-Dic61B^TRiP.HMC05696^*) |
| Dynein light chain 90F  (Dlc90F) | **GO:** dynein complex,  spermatid development  High testis expression  Male sterile [17] | ND | Y-loop gene transcription normal, sperm head scattering, sterility  (*P(PZ)Dlc90F^04091^, P(PZ)Dlc90F^05089^*) |
| Egalitarian  (Egl) | **GO:** intracellular mRNA localization, mRNA binding, mRNA transport | ND | No phenotype  (*egl^1^*, *UAS-egl^TRiP.05180^, UAS-egl^TRiP.GL01170^*) |
| Eukaryotic translation initiation factor 4A  (eIF4a) | **GO:** RNA helicase activity^11^, P granule, translation initiation | Nucleus  (*P(PTT-un)eIF4A^P02046^*) | ND |
| Eukaryotic translation initiation factor 4E1  (eIF4e1) | **GO:** chromatin organization, RNA cap binding, spermatid differentiation, translation initiation  Male sterile [18] | Cytoplasm  (*P(PTT-GC)eIF4E1^YC0001^*) | Y-loop gene transcription normal, sperm head scattering, sterility  (*UAS-eIF4E1^TRiP.HMS00969^*) |
| eukaryotic translation initiation factor 4G2  (eIF4G2) | **GO:** mRNA binding, spermatid differentiation, spermatogenesis  High testis expression  Male sterile [19, 20] | Previous study: early SC through spermatid cyst cytoplasm [20] | SC/spermatid arrest, Y-loop gene transcription mostly normal, no mRNA granules, single transcripts present in cytoplasm  (*eIF4G2^Z3-3283/BR21-37^*, gifts from Minx Fuller) |
| eukaryotic translation initiation factor 5B  (eIF5b) | **GO:** translational initiation  High testis expression | No signal  (*Mi(PT-GFSTF.0)eIF5B^MI05586-GFSTF.0^*) | ND |
| Exuperantia  (Exu) | **GO:** mRNA localization,  single-stranded RNA binding, spermatogenesis  High testis expression  Male sterile [21] | SC cytoplasm, spermatid cysts  (*GFP-exu*, Gift from Tulle Hazelrigg) | Y-loop gene transcription normal, sperm head scattering, sterility  (*exu^4^, UAS-exu^TRiP.HM05112^, UAS-exu^TRiP.GL01244^, UAS-exu^TRiP.HMS04331^*) |
| Fmr1 | **GO:** mRNA binding, mRNA transport, negative regulation of translation, sperm axoneme assembly  Male sterile [22]  K homology domains | Previous study: Cytoplasmic [23] | Y-loop gene transcription normal, sperm head scattering, sterility  (*Fmr1^Δ50M^, Fmr1^Δ113M^*) |
| Half pint  (Hfp) | **GO:** mRNA binding, poly(U) RNA binding  Male sterile [24]  RRM domains | DNA  (*P(PTT-GA)hfp^CA06961^*) | ND |
| Hephaestus  (Heph) | **GO:** mRNA binding, mRNA processing, ribonucleoprotein complex, spermatid development, translation repressor activity  Male sterile [9]  RRM domains | This study: Y-loop A & C  (*P(PTT-GC)heph^CC00664^*)  Previous study: SC cytoplasm [25] | *kl-3* and *kl-5* transcriptional defects, sperm head scattering, sterility  (*P(PZ)heph^2^*) |
| Heterogeneous nuclear ribonucleoprotein at 98DE  (Hrb98DE) | **GO:** mRNA binding, negative regulation of RNA splicing, positive regulation of translation, ribonucleoprotein complex  High testis expression  RRM domains | This study: Nucleus, SC nucleus near DNA territories  (*P(PTT-GC)Hrb98DE^ZCL0588^*  )  Previous study: Y-loop [26] | Y-loop gene transcription normal, sperm head scattering, sterility  (*UAS-Hrb98DE^TRiP.JF01249^, UAS-Hrb98DE^TRiP.HMS00342^*)  No phenotype  (*Hrb98DE^1^*) |
| IGF-II mRNA-binding protein  (Imp) | **GO:** mRNA 3’-UTR binding, spermatogenesis,  RRM domain, K homology domains | GSC – early SC cytoplasm, Y-loop, spermatid cysts  (*Mi(PT-GFSTF.2)Imp^MI05901-GFSTF.2^*) | Occasional scattering of sperm heads  (*UAS-Imp^TRiP.HMS01168^, UAS-Imp^TRiP.GL00660^, UAS-Imp^TRiP.HMC03794^*) |
| Kinesin heavy chain  (Khc) | **GO:** pole plasm oskar mRNA localization, transport along microtubule  Kinesin | SC cytoplasm, spermatid cysts (*P(αTub-Khc.GFP)R*) | ND |
| Kinesin-like protein at 3A  (Klp3a) | **GO:** microtubule motor activity  High testis expression  Kinesin | No signal  (*P(Ubi-p63E-Klp3A.GFP)1*) | ND |
| Kinesin-like protein at 59D  (Klp59D) | **GO:** microtubule motor activity, regulation of motile cilium assembly, sperm axoneme assembly  High testis expression [27]  Kinesin | Previous study: SC cytoplasm, SC cilia cap and sperm axoneme [27] | Y-loop gene transcription normal, sperm head scattering, sterility  (*UAS-Klp59D^TRiP.GL00402^, UAS-Klp59D^TRiP.HMC05692^*) |
| Kinesin-like protein at 67A  (Klp67A) | **GO:** Microtubule motor activity  High testis expression  Male sterile [28] | Nucleus  (*P(Ubi-Klp67A.GFP)1*) | ND |
| Loopin-1 | High testis expression | Previous study: Y-loop [29] | ND |
| Loquacious  (Loqs) | **GO:** double-stranded RNA binding  High testis expression  Double stranded RNA binding domains | Cytoplasmic, spermatid cysts  (*P(loqs-PA.myc)3*) | ND |
| Lost | **GO:** pole plasm mRNA localization, ribonucleoprotein complex  Male sterile [30] | Cytoplasm, strongest in SC, also spermatid cysts  (*P(PTT-GA)lost^ZCL3169^*) | Y-loop gene transcription normal, sperm head scattering, sterility  (*lost^1^*) |
| Maternal expression at 31B  (Me31B) | **GO:** cytoplasmic mRNA processing body assembly, P granule, RNA binding, RNA helicase | Cytoplasmic  (*P(PTT-GB)me31B^CB05282^*) | Y-loop gene transcription normal, sperm head scattering, sterility  (*UAS-me31B^TRiP.HMS00539^*)  No phenotype  (*UAS-me31B^TRiP.GL00695^*) |
| Muscleblind  (Mbl) | **GO:** regulation of gene expression, RNA binding  Zinc finger CCCH-type domains | This study: DNA, Y-loop  (*Mi(PT-GFSTF.0)mbl^MI00139-GFSTF.0^*)  Previous study: Y-loop [26] | No phenotype  (*UAS-mbl^TRiP.JF03264^*) |
| Mushroom-body expressed  (Mub) | **GO:** RNA binding  K homology domains | Early germ cell & cyst cell cytoplasm  (*P(PTT-GC)mub^CC01995^*) | ND |
| Non-claret disjunctional  (Ncd) | **GO:** microtubule motor activity, mRNA transport  High testis expression  Kinesin | Nucleus through early spermatids  (*P(ncd-ncd.FL.YFP)M6M1*) | ND |
| Oo18 RNA binding protein  (Orb) | **GO:** germ cell development, intracellular mRNA localization, mRNA binding, positive regulation of translation, ribonucleoprotein complex  High testis expression  RRM domains | Maybe mitochondria (*Mi(PT-GFSTF.0)orb^MI04761-GFSTF.0^*) | ND |
| Orb2 | **GO:** messenger ribonucleoprotein complex, mRNA binding, negative & positive regulation of translation, sperm axoneme assembly, sperm individualization, spermatogenesis  High testis expression  Male sterile [31]  RRM domains | Previous study: SC cytoplasm through spermatid cysts [31] | Y-loop gene transcription normal, sperm head scattering, sterility  (*PBac(WHr)orb2^36^*) |
| Outer segment (Oseg2) | **GO:** axoneme, cilium assembly, Intraciliary transport  WD40 repeat domains | No signal  *Oseg2-GFP* [32] | ND |
| Poly(A) binding protein  (pABP) | **GO:** mRNA binding, poly(A) binding, positive regulation of translation, ribonucleoprotein complex, spermatogenesis  Male sterile [33]  RRM domains | Cytoplasmic  (*pABP-GFP*, gift from Beat Suter) | Y-loop gene transcription normal, sperm head scattering, sterility  (*P(lacW)pAbp^k10109^, P(EP)pAbp^EP310^*) |
| Pontin  (Pont) | **GO:** positive regulation of gene expression  High testis expression  Axonemal dynein assembly in zebrafish/mouse [34] | ND | No phenotype detected  (*UAS-pont^TRiP.HMJ21078^*) |
| Pasilla  (Ps) | **GO:** pre-mRNA intronic binding  K homology domains | Y-loop C  (*PBac(602.P.SVS-1)ps^CPTI001063^*) [11, 26] | No phenotype detected  (*P(PZ)ps^10615^, UAS-ps^TRiP.HMS00310^, UAS-ps^TRiP.HMC04685^*) |
| Reptin  (Rept) | **GO:** negative regulation of gene expression  High testis expression  Axonemal dynein assembly in zebrafish/mouse [34] | ND | SC/spermatid arrest, possible structural defects in Y-loop gene nuclear transcripts, many single transcripts present in cytoplasm, no mRNA granules present  (*UAS-rept^TRiP.HMS00410^*) |
| Ribonuclear protein at 97D  (Rb97D) | **GO:** mRNA binding, ribonucleoprotein complex  Male sterile [35]  RRM domains | Previous study: Y-loop C [36] | Y-loop gene transcription normal, sperm head scattering, sterility  (*Rb97D^2^*) |
| Rm62 | **GO:** mRNA binding, RNA helicase  Male sterile [37] | Nucleus, around DNA in SCs  (*P(PTT-GB)Rm62^YB0077^, Mi(PT-GFSTF.2)Rm62^MI00377-GFSTF.2^*) | No phenotype  (*UAS-Rm62^TRiP.JF01385^, UAS-Rm62^TRiP.HMJ22101^, UAS-Rm62^TRiP.HMC05882^*)  Y-loop gene transcripts possibly disorganized, sperm head scattering, sterility  (*UAS-Rm62^TRiP.HMS00144^*) |
| RNA-binding protein 4  (Rbp4) | **GO:** mRNA binding, mRNA processing, single-stranded RNA binding  High testis expression  Male serility [38]  RRM domains  Translational repression in testis [20] | Previous study: SC through spermatid cytoplasm [20] | Y-loop gene transcription normal, sperm head scattering, sterility  (*rbp4^LL06910^, P(GD14281)v29116*) |
| Spermatocyte arrest  (Sa) | **GO:** regulation of gene expression, spermatid development, spermatogenesis  High testis expression  Male sterile [1]  Part of SC-specific transcriptional program^5^ [1, 2] | Previous study: SC DNA and nucleolus [39] | SC arrest, nuclear Y-loop gene transcription normal, single transcripts present in cytoplasm, no granules  (*sa^1/2^*, gifts from Minx Fuller) |
| Spt6 | **GO:** regulation of mRNA processing, transcription elongation factor complex | DNA  (*P(PTT-GA)Spt6^CA07692^*) | ND |
| Squid  (Sqd) | **GO:** intracellular mRNA localization, mRNA binding, mRNA export from nucleus, negative regulation of translation, ribonucleoprotein complex  Two RRM domains | This study: Nucleus, DNA, Y-loop  *GFP-sqd (ZCL0734)*  Previous study: Y-loop [26] | Y-loop gene transcription normal, sperm head scattering, sterility  (*UAS-sqd^TRiP.JF01248^, UAS-sqd^TRiP.JF01479^, UAS-sqd^TRiP.GL00473^, UAS-sqd^TRiP.HMC03848^*) |
| Syncrip  (Syp) | **GO:** mRNA binding, ribonucleoprotein granule  High testis expression  Three RRM domains | SC DNA, cytoplasm  (*Mi(PT-GFSTF.1)Syp^MI06413-GFSTF.1^*)  No signal  (*Mi(PT-GFSTF.1)Syp^MI02101-GFSTF.1^*) | SC arrest, Y-loop gene transcription appears normal, mRNA granule sized appeared reduced  (*UAS-Syp^TRiP.HMC04412^*) |
| Tapas | **GO:** P granule | SC nuclear periphery  (*P(PTT-GC)tapas^CC00825^*) | ND |
| Testis-specifically expressed bromodomain containing protein -1  (tBRD-1) | **GO:** spermatid differentiation, spermatogenesis  High testis expression  Male sterile [40]  Interacts with SC transcriptional program^5^ [40] | Previous study: SC nucleolus, colocalizes with sa [40] | Y-loop gene transcript normal in nucleus, kl-5 reduced in mRNA granules, scattering of sperm heads, sterility  (*UAS-tBRD-1^TRiP.HMS02321^, UAS-tBRD-1^TRiP.HMC03811^*) |
| Thoc5 | **GO:** mRNA binding, mRNA export from nucleus  Male sterile [41]  In yeast, important for transcription of genes with internal repeats [42] | Previous study: SC DNA and nucleolus [41] | No phenotype  (*UAS-thoc5^TRiP.HMC03921^*) |
| Topoisomerase 1 (Top1) |  | DNA, SC nucleolus  (*P(PTT-GC)Top1^CC01414^*) | Y-loop gene transcripts possibly disorganized in SCs, kl-5 absent from granule, kl-3 reduced in granule, sperm head scattering, sterility  (*UAS-Top1^TRiP.HMC04001^*) |
| Tudor  (Tud) | **GO:** germ cell development, intracellular mRNA localization, P granule  Six Tudor domains | Cytoplasmic  (*Tud-HA*, gift from Ruth Lehmann) | ND |
| Twin | **GO:** negative regulation of translation  High testis expression | Cytoplasm, puncta  (*Mi(PT-GFSTF.1)twin^MI07336-GFSTF.1^*) | No Phenotype  (*UAS-twin^TRiP.HMS00493^, UAS-twin^TRiP.HMS00690^*) |
| Wurstfest  (Fest) | High testis expression  Translational repression in testis [20] | Previous study: SC cytoplasm, spermatid cysts [20] | SC arrest, Y-loop gene transcription does not complete, no granules  (*fest^1^*, gift from Minx Fuller, *P(KK106182)VIE-260B*) |
| x16 splicing factor (x16) | **GO:** mRNA binding, regulation of gene expression  One RRM domain, one zinc finger CCHC-type domain | Nucleus, DNA  (*P(PTT-GB)x16^CB03248^*) | ND |

**Footnotes:**

1. Only GO terms of relevance are listed. Testis expression is noted only if the candidate is expressed more or only in the testis. Predicted relevant protein domains are noted.
2. All localization data is from this study unless otherwise noted.
3. Y-loop gene transcriptional defects as well as spermiogenesis phenotypes that resemble *kl-3* or *kl-5* RNAi are noted. RNAi efficiency was not validated for the primary screening.
4. As the Y chromosome is primarily heterochromatic, a GO term of ‘heterochromatin’ was included in this screening.

We examined Y-loop gene transcription in tMAC or tTAF mutants. While both affected Y-loop gene transcription, this may be indirect and the Y-loop gene expression program may be a unique subset of the SC transcriptional program.

1. We assume the *kl-3* and *kl-5* mRNA granules serve to delay translation and therefore genes predicted to be involved in translational regulation were of interest.
2. As the identity of the mRNA granules of *kl-3* and *kl-5* remains unknown, constituents of known RNP granules were of interest.
3. Sterility was determined by looking for sperm in the seminal vesicles. Here, no distinction is made between partial (reduced number of sperm or some empty/some full seminal vesicles) and complete sterility.

Several candidates are known to function during oogenesis to organize mRNAs within the oocyte. We hypothesized that similar mechanisms may be at play in the male to organize Y-loop gene mRNAs into RNP granules.

We analyzed several motor proteins because we hypothesized that motor proteins could be transporting Y-loop gene mRNAs to facilitate mRNA granule formation.

1. Some RNA helicases were included under the assumption that Y-loop gene RNAs may adopt complex secondary structures that may need to be resolved for proper processing.

**References:**

1. Lin TY, Viswanathan S, Wood C, Wilson PG, Wolf N, Fuller MT. Coordinate developmental control of the meiotic cell cycle and spermatid differentiation in Drosophila males. Development. 1996;122(4):1331-41. PubMed PMID: 8620860.

2. White-Cooper H, Schafer MA, Alphey LS, Fuller MT. Transcriptional and post-transcriptional control mechanisms coordinate the onset of spermatid differentiation with meiosis I in Drosophila. Development. 1998;125(1):125-34. PubMed PMID: 9389670.

3. Perezgasga L, Jiang J, Bolival B, Jr., Hiller M, Benson E, Fuller MT, et al. Regulation of transcription of meiotic cell cycle and terminal differentiation genes by the testis-specific Zn-finger protein matotopetli. Development. 2004;131(8):1691-702. doi: 10.1242/dev.01032. PubMed PMID: 15084455.

4. White-Cooper H, Leroy D, MacQueen A, Fuller MT. Transcription of meiotic cell cycle and terminal differentiation genes depends on a conserved chromatin associated protein, whose nuclear localisation is regulated. Development. 2000;127(24):5463-73. PubMed PMID: 11076766.

5. Spradling AC, Stern D, Beaton A, Rhem EJ, Laverty T, Mozden N, et al. The Berkeley Drosophila Genome Project gene disruption project: Single P-element insertions mutating 25% of vital Drosophila genes. Genetics. 1999;153(1):135-77. PubMed PMID: 10471706; PubMed Central PMCID: PMCPMC1460730.

6. Johnstone O, Deuring R, Bock R, Linder P, Fuller MT, Lasko P. Belle is a Drosophila DEAD-box protein required for viability and in the germ line. Dev Biol. 2005;277(1):92-101. doi: 10.1016/j.ydbio.2004.09.009. PubMed PMID: 15572142.

7. Gerbasi VR, Preall JB, Golden DE, Powell DW, Cummins TD, Sontheimer EJ. Blanks, a nuclear siRNA/dsRNA-binding complex component, is required for Drosophila spermiogenesis. Proceedings of the National Academy of Sciences. 2011;108(8):3204-9. doi: 10.1073/pnas.1009781108.

8. Sanders C, Smith DP. LUMP is a putative double-stranded RNA binding protein required for male fertility in Drosophila melanogaster. PLoS One. 2011;6(8):e24151. doi: 10.1371/journal.pone.0024151. PubMed PMID: 21912621; PubMed Central PMCID: PMCPMC3166160.

9. Castrillon DH, Gonczy P, Alexander S, Rawson R, Eberhart CG, Viswanathan S, et al. Toward a molecular genetic analysis of spermatogenesis in Drosophila melanogaster: characterization of male-sterile mutants generated by single P element mutagenesis. Genetics. 1993;135(2):489-505. PubMed PMID: 8244010; PubMed Central PMCID: PMC1205651.

10. Eberhart CG, Maines JZ, Wasserman SA. Meiotic cell cycle requirement for a fly homologue of human Deleted in Azoospermia. Nature. 1996;381(6585):783-5. doi: 10.1038/381783a0. PubMed PMID: 8657280.

11. Redhouse JL, Mozziconacci J, White RA. Co-transcriptional architecture in a Y loop in Drosophila melanogaster. Chromosoma. 2011;120(4):399-407. doi: 10.1007/s00412-011-0321-1. PubMed PMID: 21556802.

12. Cox RT, Spradling AC. Clueless, a conserved Drosophila gene required for mitochondrial subcellular localization, interacts genetically with parkin. Dis Model Mech. 2009;2(9-10):490-9. doi: 10.1242/dmm.002378. PubMed PMID: 19638420; PubMed Central PMCID: PMCPMC2737057.

13. Ghosh-Roy A, Kulkarni M, Kumar V, Shirolikar S, Ray K. Cytoplasmic dynein-dynactin complex is required for spermatid growth but not axoneme assembly in Drosophila. Mol Biol Cell. 2004;15(5):2470-83. doi: 10.1091/mbc.e03-11-0848. PubMed PMID: 15020714; PubMed Central PMCID: PMCPMC404038.

14. Ceprani F, Raffa GD, Petrucci R, Piergentili R. Autosomal mutations affecting Y chromosome loops in Drosophila melanogaster. BMC Genet. 2008;9:32. doi: 10.1186/1471-2156-9-32. PubMed PMID: 18405358; PubMed Central PMCID: PMC2386818.

15. Akanksha, Mallik M, Fatima R, Lakhotia SC. The hsromega(05241) allele of the noncoding hsromega gene of Drosophila melanogaster is not responsible for male sterility as reported earlier. J Genet. 2008;87(1):87-90. PubMed PMID: 18560179.

16. Fatima R. Drosophila Dynein intermediate chain gene, Dic61B, is required for spermatogenesis. PLoS One. 2011;6(12):e27822. doi: 10.1371/journal.pone.0027822. PubMed PMID: 22145020; PubMed Central PMCID: PMCPMC3228723.

17. Caggese C, Moschetti R, Ragone G, Barsanti P, Caizzi R. dtctex-1, the Drosophila melanogaster homolog of a putative murine t-complex distorter encoding a dynein light chain, is required for production of functional sperm. Mol Genet Genomics. 2001;265(3):436-44. PubMed PMID: 11405626.

18. Ghosh S, Lasko P. Loss-of-function analysis reveals distinct requirements of the translation initiation factors eIF4E, eIF4E-3, eIF4G and eIF4G2 in Drosophila spermatogenesis. PLoS One. 2015;10(4):e0122519. doi: 10.1371/journal.pone.0122519. PubMed PMID: 25849588; PubMed Central PMCID: PMCPMC4388691.

19. Franklin-Dumont TM, Chatterjee C, Wasserman SA, Dinardo S. A novel eIF4G homolog, Off-schedule, couples translational control to meiosis and differentiation in Drosophila spermatocytes. Development. 2007;134(15):2851-61. doi: 10.1242/dev.003517. PubMed PMID: 17611222.

20. Baker CC, Fuller MT. Translational control of meiotic cell cycle progression and spermatid differentiation in male germ cells by a novel eIF4G homolog. Development. 2007;134(15):2863-9. doi: 10.1242/dev.003764. PubMed PMID: 17611220.

21. Hazelrigg T, Watkins WS, Marcey D, Tu C, Karow M, Lin XR. The exuperantia gene is required for Drosophila spermatogenesis as well as anteroposterior polarity of the developing oocyte, and encodes overlapping sex-specific transcripts. Genetics. 1990;126(3):607-17. PubMed PMID: 2249760; PubMed Central PMCID: PMCPMC1204216.

22. Zhang YQ, Matthies HJ, Mancuso J, Andrews HK, Woodruff E, 3rd, Friedman D, et al. The Drosophila fragile X-related gene regulates axoneme differentiation during spermatogenesis. Dev Biol. 2004;270(2):290-307. doi: 10.1016/j.ydbio.2004.02.010. PubMed PMID: 15183715.

23. Bozzetti MP, Specchia V, Cattenoz PB, Laneve P, Geusa A, Sahin HB, et al. The Drosophila fragile X mental retardation protein participates in the piRNA pathway. J Cell Sci. 2015;128(11):2070-84. doi: 10.1242/jcs.161810. PubMed PMID: 25908854.

24. Van Buskirk C, Schupbach T. Half pint regulates alternative splice site selection in Drosophila. Dev Cell. 2002;2(3):343-53. PubMed PMID: 11879639.

25. Robida M, Sridharan V, Morgan S, Rao T, Singh R. Drosophila polypyrimidine tract-binding protein is necessary for spermatid individualization. Proc Natl Acad Sci U S A. 2010;107(28):12570-5. Epub 2010/07/10. doi: 10.1073/pnas.1007935107. PubMed PMID: 20616016; PubMed Central PMCID: PMCPMC2906604.

26. Lowe N, Rees JS, Roote J, Ryder E, Armean IM, Johnson G, et al. Analysis of the expression patterns, subcellular localisations and interaction partners of Drosophila proteins using a pigP protein trap library. Development. 2014;141(20):3994-4005. doi: 10.1242/dev.111054. PubMed PMID: 25294943; PubMed Central PMCID: PMCPMC4197710.

27. Vieillard J, Paschaki M, Duteyrat JL, Augiere C, Cortier E, Lapart JA, et al. Transition zone assembly and its contribution to axoneme formation in Drosophila male germ cells. J Cell Biol. 2016;214(7):875-89. doi: 10.1083/jcb.201603086. PubMed PMID: 27646273; PubMed Central PMCID: PMCPMC5037411.

28. Gandhi R, Bonaccorsi S, Wentworth D, Doxsey S, Gatti M, Pereira A. The Drosophila kinesin-like protein KLP67A is essential for mitotic and male meiotic spindle assembly. Mol Biol Cell. 2004;15(1):121-31. doi: 10.1091/mbc.e03-05-0342. PubMed PMID: 13679514; PubMed Central PMCID: PMCPMC307533.

29. Piergentili R. Evolutionary conservation of lampbrush-like loops in drosophilids. BMC Cell Biol. 2007;8:35. doi: 10.1186/1471-2121-8-35. PubMed PMID: 17697358; PubMed Central PMCID: PMC1978495.

30. Sinsimer KS, Jain RA, Chatterjee S, Gavis ER. A late phase of germ plasm accumulation during Drosophila oogenesis requires lost and rumpelstiltskin. Development. 2011;138(16):3431-40. doi: 10.1242/dev.065029. PubMed PMID: 21752933; PubMed Central PMCID: PMCPMC3143563.

31. Xu S, Hafer N, Agunwamba B, Schedl P. The CPEB protein Orb2 has multiple functions during spermatogenesis in Drosophila melanogaster. PLoS Genet. 2012;8(11):e1003079. doi: 10.1371/journal.pgen.1003079. PubMed PMID: 23209437; PubMed Central PMCID: PMCPMC3510050.

32. Avidor-Reiss T, Maer AM, Koundakjian E, Polyanovsky A, Keil T, Subramaniam S, et al. Decoding cilia function: defining specialized genes required for compartmentalized cilia biogenesis. Cell. 2004;117(4):527-39. PubMed PMID: 15137945.

33. Blagden SP, Gatt MK, Archambault V, Lada K, Ichihara K, Lilley KS, et al. Drosophila Larp associates with poly(A)-binding protein and is required for male fertility and syncytial embryo development. Dev Biol. 2009;334(1):186-97. doi: 10.1016/j.ydbio.2009.07.016. PubMed PMID: 19631203.

34. Li Y, Zhao L, Yuan S, Zhang J, Sun Z. Axonemal dynein assembly requires the R2TP complex component Pontin. Development. 2017;144(24):4684-93. doi: 10.1242/dev.152314. PubMed PMID: 29113992; PubMed Central PMCID: PMCPMC5769618.

35. Karsch-Mizrachi I, Haynes SR. The Rb97D gene encodes a potential RNA-binding protein required for spermatogenesis in Drosophila. Nucleic Acids Res. 1993;21(9):2229-35. PubMed PMID: 8502565; PubMed Central PMCID: PMCPMC309489.

36. Heatwole VM, Haynes SR. Association of RB97D, an RRM protein required for male fertility, with a Y chromosome lampbrush loop in Drosophila spermatocytes. Chromosoma. 1996;105(5):285-92. PubMed PMID: 8939821.

37. Buszczak M, Paterno S, Lighthouse D, Bachman J, Planck J, Owen S, et al. The carnegie protein trap library: a versatile tool for Drosophila developmental studies. Genetics. 2007;175(3):1505-31. doi: 10.1534/genetics.106.065961. PubMed PMID: 17194782; PubMed Central PMCID: PMCPMC1840051.

38. Haynes SR, Cooper MT, Pype S, Stolow DT. Involvement of a tissue-specific RNA recognition motif protein in Drosophila spermatogenesis. Mol Cell Biol. 1997;17(5):2708-15. PubMed PMID: 9111341; PubMed Central PMCID: PMCPMC232121.

39. Chen X, Hiller M, Sancak Y, Fuller MT. Tissue-specific TAFs counteract Polycomb to turn on terminal differentiation. Science. 2005;310(5749):869-72. doi: 10.1126/science.1118101. PubMed PMID: 16272126.

40. Leser K, Awe S, Barckmann B, Renkawitz-Pohl R, Rathke C. The bromodomain-containing protein tBRD-1 is specifically expressed in spermatocytes and is essential for male fertility. Biol Open. 2012;1(6):597-606. doi: 10.1242/bio.20121255. PubMed PMID: 23213453; PubMed Central PMCID: PMCPMC3509448.

41. Moon S, Cho B, Min SH, Lee D, Chung YD. The THO complex is required for nucleolar integrity in Drosophila spermatocytes. Development. 2011;138(17):3835-45. doi: 10.1242/dev.056945. PubMed PMID: 21828100.

42. Voynov V, Verstrepen KJ, Jansen A, Runner VM, Buratowski S, Fink GR. Genes with internal repeats require the THO complex for transcription. Proc Natl Acad Sci U S A. 2006;103(39):14423-8. doi: 10.1073/pnas.0606546103. PubMed PMID: 16983072; PubMed Central PMCID: PMCPMC1599979.
