# Supplementary material for: Satellite DNA-containing gigantic introns in a unique gene expression program during Drosophila spermatogenesis
Source: PLoS Genet. 2019 May 9;15(5):e1008028. doi: 10.1371/journal.pgen.1008028 (PMC6508621; doi:10.1371/journal.pgen.1008028)
Supplement: S3 File — (DOCX) [file pgen.1008028.s006.docx]

**S3 File: RT-qPCR primers**

| **Primer Name** | **5’-Sequence-3’** |
| --- | --- |
| Gapdh-qF | TAAATTCGACTCGACTCACGGT |
| Gapdh-qR | CTCCACCACATACTCGGCTC |
| Kl-3_early_normal_exons2-3_qF | TTGGGATCCCTTATACCGttcttc* |
| Kl-3_early_normal_exon3_qR | CCATAAGACCTGTAACGTTGACAG |
| Kl-3_early_large_exon1_qF | CCCGAGCATTTAATAACCACAAG |
| Kl-3_early_large_exon2_qR | AACGGACATTATCCTTAGCTTCA |
| Kl-3_middle_normal_exons6-7_qF | GGCGTGTTACTGTCGatgaa* |
| Kl-3_middle_normal_exon7_qR | CACGCTGAAATTCTTCCATGTC |
| Kl-3_middle_large_exon5_qF | GCTGGATCTAAGAGGTCATTGG |
| Kl-3_middle_large_exon6_qR | GGCTGAATGTAACACCCGTTAT |
| Kl-3_late_normal_exons14-15_qF | TATGTCCATTCAACCTAAAGaatcgtc* |
| Kl-3_late_normal_exon15_qR | CCCATTGCAATTAGATGCTGTT |
| Kl-3_late_large_exon15_qF | GCCACGAGCTCGATGAATA |
| Kl-3_late_large_exon16_qR | AGTACCTTCAACGGCAAGAA |
| Kl-5_early_normal_exons7-8_qF | CACGAACTTTACGAATATCCacttt* |
| Kl-5_early_normal_exon8_qR | CCTGCCAGCACTCAACA |
| Kl-5_early_large_exon1_qF | ATGCGTCTTAAGCTGGATAAGT |
| Kl-5_early_large_exon2_qR | TGTCCACCGGAATTGATTGT |
| Kl-5_middle_normal_exons10-11_qF | TGAATCCTTACAGCTTCTATGatgag* |
| Kl-5_middle_normal_exon11_qR | TTTGCCATGGACACGCA |
| Kl-5_middle_large_exon12_qF | CGAGCAATCAAATCGGTTCTTG |
| Kl-5_middle_large_exon13_qR | ACACTGGTACATCATCGGTAAC |
| Kl-5_late_normal_exons15-16_qF | GACCCTCAACTtcagggaataaa* |
| Kl-5_late_normal_exon16_qR | AGTAATACACTTCCATTACTGACTG |
| Kl-5_late_large_exon16_qF | GCCTCTCGATAGAATGTGTCTT |
| Kl-5_late_large_exon17_qR | TTTCATGTCCCATCGTGCT |

*A change in case represents the bridge between the two exons indicated in the primer name.
